# Effects of cannabidiol (CBD) treatment on age-related cognitive decline in C57 mice

**DOI:** 10.3389/fnagi.2025.1567650

**Published:** 2025-05-09

**Authors:** Behroo Mirza Agha, Merrin Monteith, Jarem Earl, Keona Ganske, Tina Kaloa, Kelan J. McDonald, Abigail G. Nixon, Maleeha Panjwani, Danika Robinson, Valeria Rusnak, Majid H. Mohajerani, Igor Kovalchuk, Robert J. Sutherland, Nancy S. Hong, Robert J. McDonald

**Affiliations:** ^1^Canadian Centre for Behavioural Neuroscience, Department of Neuroscience, University of Lethbridge, Lethbridge, AB, Canada; ^2^Douglas Research Centre, Department of Psychiatry, McGill University, Montréal, QC, Canada; ^3^Department of Biological Sciences, University of Lethbridge, Lethbridge, AB, Canada

**Keywords:** aging, age-related cognitive decline, cannabidiol (CBD), learning and memory, hippocampus, prefrontal cortex, inflammation, acetylcholine

## Abstract

Aging is associated with cognitive decline, and currently, there are no approved medications that can prevent these impairments. Recently, cannabinoids derived from *Cannabis sativa* have emerged as promising therapeutic compounds with neuroprotective, anti-inflammatory, and cognitive-enhancing properties. Despite their benefits, further research is needed to fully understand their efficacy across various conditions. This study investigates the effects of cannabidiol (CBD) on memory impairment and brain inflammation in aging mice. Fourteen-month-old C57 mice were administered CBD orally for 7 months and subsequently evaluated between 19 and 21 months of age using behavioral tasks that are sensitive to dysfunction of the perirhinal cortex, hippocampus, amygdala, and various brain regions that are crucial for motor control and coordination. The findings of this study indicate that CBD reduces inflammatory response in the brain and improves cognitive decline associated with aging.

## Introduction

Aging is inevitable, and it is a universal process that brings about biological, psychological, and physiological changes in individuals (Burke and Barnes, 2006). The brain also undergoes physical and neurobiological changes that can lead to impaired cognition. These changes are considered a normal part of the aging process (Murman, 2015; Peters, 2006). In contrast, Alzheimer's disease (AD) is characterized behaviorally by progressive memory impairment, confusion, loss of cognitive abilities, and various brain changes, including abnormal protein accumulation [amyloid-beta and tau (Braak et al., 1994; Braak and Braak, 1991)], inflammation (Akiyama, 2000), and neuron and synapse loss (Tzioras et al., 2022).

Normal cognitive aging is different from AD, and the anatomical and morphological changes in the brain due to aging differ from those observed in AD in both humans and animals. In aging humans and rodents, a decrease in hippocampus (HPC) volume is observed, likely due in part to a decrease in HPC neurogenesis or shrinkage of neurons, which in turn can contribute to deficits in HPC-dependent memory tasks. Notably, significant cell death, reduction in dendritic length or branching, and decrease in spine density are not typical features of the normal aging process (Burke and Barnes, 2006; Driscoll et al., 2006; Driscoll and Sutherland, 2005; Rapp and Gallagher, 1996; Rasmussen et al., 1996; Raz, 2000; West et al., 1994). Similarly, cognitive changes that occur during normal aging differ from those observed in AD in both humans and animals. For example, both young and old rats can learn a cognitive task in the same number of trials. However, older rats tend to shift away from using HPC-dependent strategies and instead opt for alternative approaches. Consequently, they show deficits in HPC-dependent spatial learning and memory in tasks such as the Morris water task (MWT) and Barnes maze (Barnes, 1979; Foster et al., 2012; Gage et al., 1984). Similar to rats, changes in spatial strategy are also observed in older rhesus macaques and humans (Driscoll and Sutherland, 2005; Rapp et al., 1997).

To date, several approaches that can prevent dementia in both humans and animal models have been investigated. These approaches include pharmacological and non-pharmacological interventions, such as lifestyle modifications, cognitive training and mental stimulation, and sleep improvement (Homolak et al., 2018; Li et al., 2023; Stefaniak et al., 2022; Yu et al., 2021). In recent years, cannabinoids, active compounds found in the plant *Cannabis sativa*, have emerged as novel therapeutic compounds to treat a variety of neurodegenerative diseases (Pagano et al., 2022; Ramírez et al., 2005). Given their recognized neuroprotective properties (Ben-Shabat et al., 1998; Marsicano et al., 2002) and anti-inflammation effects (Burstein, 2015; Nagarkatti et al., 2009), cannabinoids have also been proposed to enhance cognitive function (Bachmeier et al., 2013). Nevertheless, numerous review studies on the effect of cannabinoids on dementia have concluded that the available evidence is inconclusive and insufficient (Bilbao and Spanagel, 2022; Bosnjak Kuharic et al., 2021; Peprah and Mccormack, 2019). Although cannabinoids may have a minimal impact on familial AD, in which more aggressive brain changes and pathology are observed than in the sporadic form of AD (McDonald, 2002; McDonald et al., 2010) or normal aging, they may potentially offer symptomatic relief to individuals experiencing normal cognitive decline. There are three major components in *Cannabis sativa*: phytocannabinoids, terpenes, and flavonoids. Phytocannabinoids are cannabinoids that are naturally found in the cannabis plant. Delta-9-tetrahydrocannabinol (THC) and cannabidiol (CBD) are the major constituents of cannabis that are of medical interest. THC is the primary psychoactive component, and CBD is the primary non-psychotomimetic component (Wang et al., 2022). CBD modulates the endocannabinoid system chiefly through indirect mechanisms. It acts as a negative allosteric modulator at the CB1 receptor (Laprairie et al., 2015), reducing the receptor's response despite increased anandamide. The action of CBD on the CB2 receptor is more complex and context-dependent, with some studies suggesting weak inverse agonism or allosteric modulation (McPartland et al., 2015; Pertwee, 2008). In addition, CBD also interacts with various neurotransmitter systems (Abame et al., 2021; Chrestia et al., 2022; McPartland et al., 2015).

The present study investigated the effect of CBD on memory decline and brain pathology in aging mice. In this study, 14-month-old C57 mice were given either CBD or a vehicle orally for 7 months and were subjected to behavioral tasks at the age of 19–21 months, such as novel object recognition (NOR), MWT, discriminative fear conditioning to context (DFCTC), and balance beam (BB), that are sensitive to the dysfunction of networks centered on the perirhinal cortex (PRh), HPC, amygdala, and motor coordination, respectively. We hypothesized that aged mice that received CBD would perform better in all these tasks compared to those that received vehicle. We also quantified the volume of HPC and inflammation markers (astrocytes and microglia) in the HPC and medial prefrontal cortex (mPFC). Finally, we assessed the status of cholinergic neurons in the medial septum (MS)/diagonal band (DB) that project to the HPC and neocortex.

## Methods

### Ethics approval

All experiments were carried out in accordance with Canadian Council of Animal Care and approved by the University of Lethbridge Animal Welfare Committee.

### Animals

In this study, we used 19 (15 male and 4 female) C57BL/6 mice (14 months of age at the beginning of the study), weighing 27–44 g [28–44 g (CBD group) and 27–37 g (vehicle group)], bred and raised at the Canadian Centre for Behavioural Neuroscience Vivarium, University of Lethbridge. The animals were housed in pairs under a 12:12 h light/dark cycle, with the lights on at 7:30 a.m. and temperature set at 22°C. All mice had *ad libitum* access to food and water. All treatments, training, and testing were carried out during the light phase of the cycle at the same time every day.

### Experimental design

**Figure 1:**
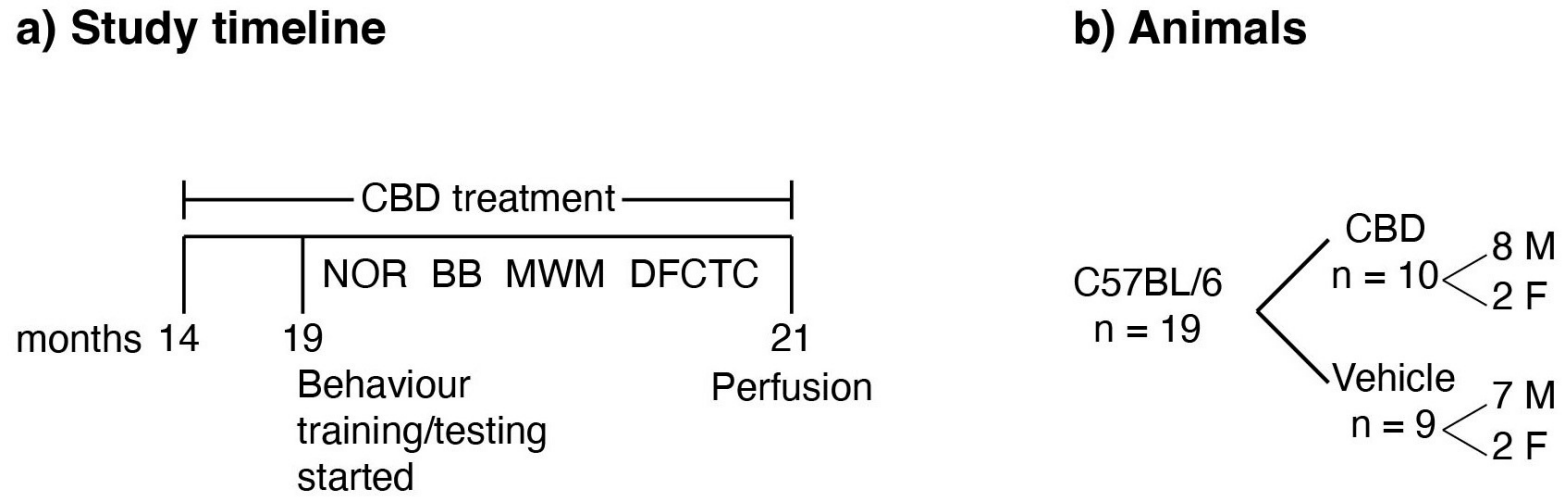
Study timeline and animal groups. **(a)** Time course of treatment and behavioral training/tests. Behavioral tests included novel object recognition (NOR), balance beam (BB), Morris water task (MWT), and discriminative fear conditioning to context (DFCTC). During behavioral training/testing, treatment with CBD and vehicle was continued daily. **(b)** The number and sex of mice per group. The CBD group received CBD in grapeseed oil and Nutella, whereas the vehicle group received only grapeseed oil and Nutella.

Mice were randomly assigned to two groups: CBD (*n* = 10, 8 male and 2 female mice) and vehicle (*n* = 9, 7 male and 2 female mice), and they were subjected to identical handling, testing, and treatment procedures. Starting at 14 months of age, for the following 7 months, they were daily treated with either CBD or vehicle in Nutella. Behavioral testing in NOR, BB, MWT, and DFCTC tasks was started when the mice were 19 months old and lasted for 2 months. All behavioral tasks were carried out during the light cycle at the same time every day. The day after the last day of testing, mice were perfused at 21 months of age, and their brains were extracted for further histological analysis (Figure 1).

### Drugs, preparation, and administration

The CBD powder (I.K. lab) was stored in a −20°C freezer before dilution. All dilutions were carried out in a biosafety cabinet fume hood. To prevent product contamination, instruments and materials were sprayed with 100% ethanol and placed under UV light for 15 min except for CBD and oil (PC 100% pure grapeseed oil, No Frills). The oil was filtered using a sterile 0.22-micron syringe filter (FroggaBio, Canada). A stock solution of oil and CBD powder (20 mg/ml) was heated on a rotating plate to 40°C to allow the powder to dissolve. The solution was aliquoted in Eppendorf tubes and stored at −20°C. While the CBD oil was being used, it was stored at 4°C for 3–4 days.

The treatment was carried out daily by mixing CBD or vehicle in 0.25 g of Nutella. During the treatment, mice were transferred from their housing cages to individual holding cages, which contained a water bottle and a weigh boat with the Nutella/CBD grapeseed oil mix for the CBD group and Nutella/grapeseed oil only for the vehicle group. Mice were weighed every other day to determine the amount of CBD/vehicle (20 ml/kg) they would receive. Holding cages were cleaned daily with Virkon, and water bottles were replaced weekly. Mice were placed in the holding cages for a maximum of 1 h or until every mouse had finished eating its Nutella mix. The dose followed in this study was based on pilot studies assessing dose–response curves that were conducted in our laboratory (Nixon et al., 2022) and previous research (Esposito et al., 2007, 2011; Hayakawa et al., 2008; Martín-Moreno et al., 2011).

### Behavioral tasks

#### Novel object recognition

This task is based on the demonstration that mice have an innate preference to explore novel items without requiring external motivation, reward, or punishment. Research has shown that this task is dependent on the PRh (Kealy and Commins, 2011). When mice that become familiar with two similar objects during the training day are provided with a novel object along with a familiar object on a subsequent test day in a familiar environment, they will spend more time exploring the novel object. This pattern of behavior indicates that mice formed memories of the objects during training and noticed the presence of a novel object during testing (Antunes and Biala, 2011). The NOR test was carried out as described in previous studies (Mehla et al., 2017, Mehla et al., 2019; Vogel-Ciernia and Wood, 2014). Briefly, the mice were transferred from their housing room to the experimental room and familiarized with a white square box (50 cm width × 50 cm length × 30 cm height) with a bedding-covered floor by placing them in this box for 10 min daily for 3 days. Training was conducted 24 h after the last habituation day. The mice were placed in the box and allowed to explore two similar objects for 10 min. A test session (test 1) was conducted 24 h after training, in which one of the familiar objects was replaced with a novel object with a different geometry and texture. Mice were given 5 min to explore the familiar and novel objects. A second test session (test 2) was conducted 24 h later, in which the new object from the previous day became the familiar object and a novel object was placed in the box. The mice were allowed 5 min to explore the objects. A retention test was conducted 1 month later, in which mice were placed in the box with the familiar object from test one and a completely new object that they had never interacted with previously. To account for side preference among mice, on all test days, half of the mice in each group had the new object placed on the right and other half had it placed on the left. During habituation, training, testing, and retention, mice were individually placed in the box, and after removing each mouse from the testing box, feces were removed and the corncob bedding was stirred to equally distribute any odor cues. In addition, the objects were wiped with 70% isopropyl alcohol to mask odor cues after each mouse and allowed to completely dry before placing the next mouse in the box. An overhead video camera (Panasonic HDC-SDT750, China) recorded the mice's behavior for further analysis. The investigation ratio (IR) for the novel object was calculated by dividing the time spent by mice exploring the novel object by the total exploration time for both objects for each group.

#### Balance beam

This test serves as an assessment tool to detect age-related changes in balance and motor coordination, given that physical decline and reduced mobility are common characteristics of aging (Orenduff et al., 2021). In the BB task, mice were required to walk on an elevated narrow aluminum beam (1 cm in diameter, 100 cm long, and 50 cm above a foam pad) to reach an enclosed escape box. Initially, mice were trained by placing them at distances of 10 cm, 50 cm, and 90 cm from the escape box and were required to complete one successful crossing or a maximum of six trials at each distance. On the second training day, mice were placed 90 cm from the escape box and were given a maximum of six trials or until they completed three successful crossings. On the third day (test day), mice were placed 90 cm from the escape box and given three trials. During training and testing, the beam and the escape box were cleaned with 70% isopropyl alcohol. Furthermore, the mean latency (in seconds) was scored manually.

#### Morris water task

This task is widely recognized and used to study hippocampal-dependent spatial learning and memory (Morris, 1981; Sutherland et al., 1982). Rodents learn to orient themselves using distal visual cues to locate the hidden platform. The MWT was conducted as reported in previous studies (Mehla et al., 2017, Mehla et al., 2019). In brief, the pool (154 cm in diameter, 50 cm deep) was filled with water to a depth of 40 cm and made opaque by adding non-toxic white paint, and water temperature was maintained at 22 ± 1°C. The pool was divided into four quadrants. A circular platform (11 cm in diameter) was kept 0.5–1 cm below the water surface in one of the quadrants. Three distinct visual cues were placed around the pool to help mice navigate to the platform position. For 8 days of the acquisition phase, each mouse received four distributed training trials from each quadrant randomly. The trial was completed once the mouse found the platform or 60 s had elapsed. If the mouse failed to find the platform on a given trial, it was guided to find the platform. After finding the platform or aided placement, the mouse remained on the platform for 10 s. Data were recorded using an automated tracking system (HVS Image Hampton, UK) and analyzed using wtr2100 software (Actimetrics). The latency to find the platform was used as an indicator of the spatial learning ability of mice. The swimming speed of mice was analyzed to rule out the involvement of motor function as a confounding factor. The thigmotaxis behavior of mice, as an indicator of anxiety levels, during the acquisition phase was also analyzed as described in previous studies (Mehla et al., 2023, 2022). A single probe trial was conducted on the 9^th^ day to evaluate spatial memory performance. The data collected during the probe trial were analyzed, and the time spent by mice in the target quadrant and the average time spent in the other three quadrants were measured. Furthermore, average proximity and annulus crossing were analyzed to assess the spatial accuracy of the search for the platform during the probe trial.

#### Discriminative fear conditioning to context

In this task, mice learn to associate a specific environment with a fear-inducing stimulus, which is useful in the investigation of memory and fear responses (Antoniadis and McDonald, 1999; Antoniadis, 2000). In the DFCTC test, both groups underwent a pre-exposure day during which they explored two different contexts, each with distinct shapes, colors, and scent cues (a white square with amyl acetate scent and a stripped black-and-white triangle with a Vicks scent). These contexts were connected by an alleyway, allowing mice to explore both contexts for 10 min. The amount of time each mouse spent in each context was used to determine the paired context, where they would receive a foot shock, and the unpaired context, where nothing would happen. During the training days (days 1–8), mice were alternatively placed in the paired and unpaired contexts (counterbalanced) for 5 min every day, spending a total of 4 days in each context. In the paired context, mice received three 2-s foot shocks (0.5 mA) at the second, third, and fourth minutes of the session. On days 9 and 10, mice were placed in their assigned context and filmed for 5 min to assess freezing behavior. On day 11 (preference test), the two contexts were connected by the alleyway, and mice were filmed for 10 min to determine the time spent in each context. The pre-exposure, unpaired context training test, and preference test were conducted in room A, whereas only the paired context training was carried out in room B.

### Histology

After the completion of behavioral tests, mice were anesthetized and perfused transcardially with 1 × phosphate buffered saline (PBS) followed by 4% paraformaldehyde (PFA) in 1 × PBS. The brain was removed, post-fixed in 4% PFA overnight at 4°C, and then cryoprotected in a 30% sucrose solution in 1 × PBS and 0.02% sodium azide. The brain was sectioned coronally at 40 μm using a freezing microtome. One brain in the aged CBD group was poorly perfused, and therefore, only Nissl staining could be performed for this brain, resulting in 18 brains being used for immunohistochemistry (IHC) analysis. Nissl staining and IHC procedures for identifying astrocytes [glial fibrillary acidic protein (GFAP)] and microglia [ionized calcium-binding adaptor molecule 1 (Iba1)] were carried out on a subset of sections, with every ninth section being mounted on a charged Superfrost Plus Microscope Slide (VWR). In IHC procedures, choline acetyltransferase (ChAT) was used to stain cholinergic neurons, and every fifth section was mounted. For Nissl staining, sections were stained with cresyl violet and cover-slipped using Permount (Fisher Scientific). For GFAP, Iba1, and ChAT, slides were washed in Tris-buffered saline (TBS) and blocked in a solution containing 3% goat serum and 0.3% Triton-X in TBS for 2 h. The slides were then incubated with primary antibodies in TBS with 0.3% Triton-X at room temperature in a dark humid chamber for 24 h. The following primary antibodies were used: rabbit anti-GFAP (polyclonal, Ab7260, Abcam, 1:2,000); rabbit anti-Iba1 (SAF4318, 019-19741, Wako, 1:1,000); and rabbit anti-ChAT (monoclonal, Ab178850, Abcam, 1:500). After the first incubation, the slides were washed three times for 10 min and then incubated with the secondary antibody, goat anti-rabbit-alexa-594 (IgG [H+L], Invitrogen, 1:1,000), in TBS with 0.3% Triton-X for 24 h for GFAP, Iba1, and ChAT. The slides were again washed three times for 10 min. Then, Iba1 sections were incubated with DAPI (1:2,000 of the 20 ug/ml stock in TBS) for 1 h. After that, the slides were washed once for 10 min. Then, the sections on the slides were covered with Vectashield H-1000 (Vector Laboratories), cover-slipped, and later sealed with nail polish. Finally, whole slides were imaged using Nanozoomer (2.0-RS, Hamamatsu, Japan) with 40 × objective magnification.

### Histological analysis

The HPC volume in each mouse was estimated following the Cavalieri method utilizing a set of 12 cross-sections of the hippocampal area, starting from −0.94 mm and terminating at -−3.08 mm relative to Bregma (Paxinos and Franklin, 2019) using a light microscope with 10 × objective magnification. GFAP and Iba1 images were analyzed using ImageJ (NIH) and Ilastik (WWW download) (Berg et al., 2019) software.

### Statistical analysis

Statistical analyses were conducted using SPSS (v.22.0) statistical software package. Results were presented as mean ± SE. A repeated-measures ANOVA was used to determine statistically significant differences across days during the acquisition phase of MWT, between the target and the average of other quadrants during the probe trial of MWT, and for the DFCTC task. An independent-sample *t-*test was used to measure significant differences in the NOR, BB, proximity and annulus crossing in the MWT, HPC volume, GFAP and Iba1 percent coverage, and ChAT counts. Bonferroni correction was used for *post-hoc* comparison. A *p*-value of < 0.05 was considered statistically significant.

## Results

### CBD improved long-term retention on the NOR

**Figure 2:**
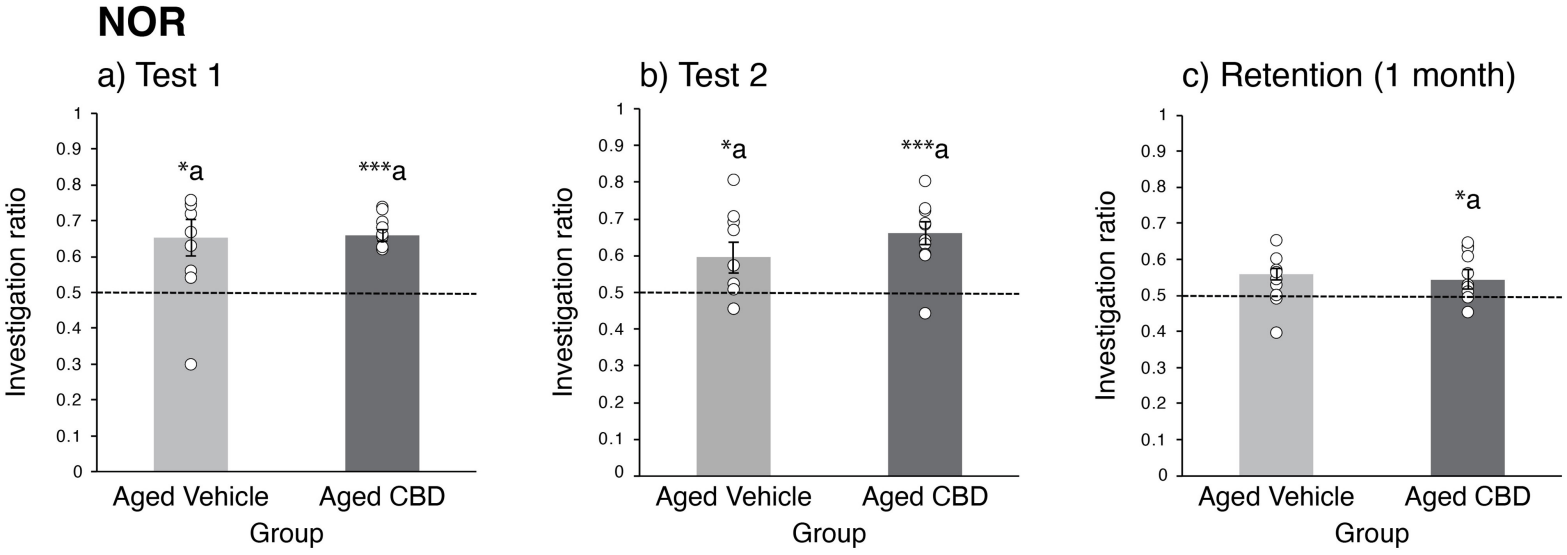
Effect of CBD on learning and memory function of 19-month-old C57BL/6 mice in the novel object recognition (NOR) task. **(a)** Investigation ratio for vehicle and CBD groups in test 1, in which a familiar and a new object are used. **(b)** Investigation ratio for vehicle and CBD groups in test 2, in which the new object in test 1 has become familiar and another new object is used. **(c)** Investigation ratio for vehicle and CBD groups 1 month later with the familiar object in test 1 and a completely novel object. *P < 0.05 and ***P < 0.001 are considered statistically significant. a—as compared to 0.5 investigation ratio (chance level). Vehicle group (*n* = 9; 7 male and 2 female), CBD group (*n* = 10; 8 male and 2 female).

During the training session, no significant difference was observed in exploration time for identical objects A1 and A2 in vehicle and CBD groups. The IR of the novel object in both groups is shown in Figure 2. During tests 1 and 2, a two-tailed one-sample *t*-test revealed that all mice explored the novel object significantly more than the familiar object, as determined by the IR value higher than 0.5 for the aged CBD group for both test 1 and test 2 [t_(9)_ = 14.630, *P* < 0.001; and t_(9)_ = 4.976, *P* < 0.001, respectively] and for the aged vehicle group for test 1 and test 2 [t_(8)_ = 2.646, *P* = 0.029; and t _(8)_ = 2.389, *P* = 0.044, respectively]. During the one-month retention test, mice that received CBD explored the novel object significantly more than mice in the vehicle group, as determined by the IR value higher than 0.5 [t_(9)_ = 2.876, *P* = 0.018 for the aged CBD group; and t_(8)_ = 1.669, *P* = 0.134 for the aged vehicle group].

### CBD had no effect on motor balance and coordination

**Figure 3:**
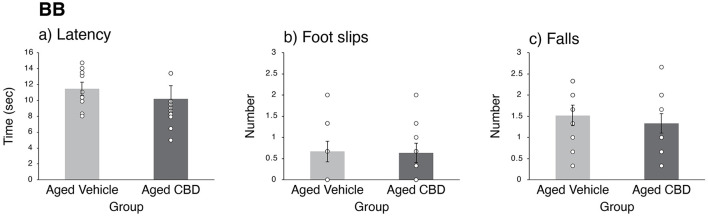
Effect of CBD on motor balance and coordination of 19-month-old C57BL/6 mice in the balance beam (BB) task. **(a)** Average (mean ± SEM) latency to cross the beam in three trials for vehicle and CBD groups. **(b)** Average (mean ± SEM) number of foot slips in three trials for vehicle and CBD groups. **(c)** Average (mean ± SEM) number of falls in three trials for vehicle and CBD groups. Vehicle group (*n* = 9; 7 male and 2 female), CBD group (*n* = 10; 8 male and 2 female).

The mean latency, number of foot slips, and number of falls in the BB test in aged vehicle and CBD groups are shown in Figure 3. The latency to cross the beam, number of foot slips, and number of falls were not significantly different between the vehicle and CBD groups, as confirmed by independent sample *t*-tests [t_(17)_ = 0.104, *P* = 0.919; t_(16.745)_ = 0.556, *P* = 0.585; and t_(17)_ = 0.653, *P* = 0.522, respectively].

### CBD improved spatial memory consolidation

**Figure 4:**
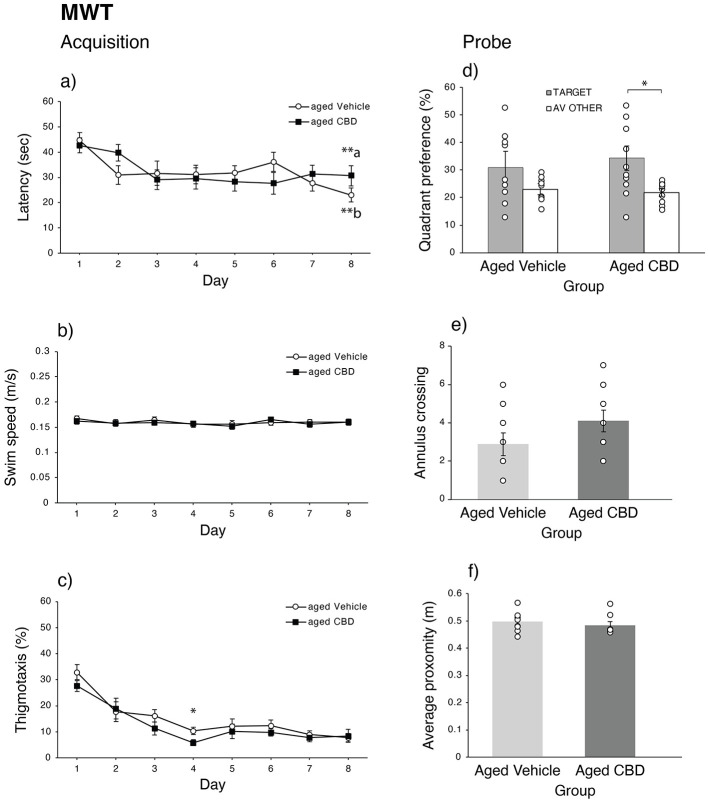
Effect of CBD on spatial learning and memory function of 19-month-old C57BL/6 mice in the Morris water task (MWT). **(a)** Mean latency (mean ± SEM) to find the hidden platform during the acquisition phase for vehicle and CBD groups. **(b)** Mean swimming speed (mean ± SEM) of mice during the acquisition phase for vehicle and CBD groups. **(c)** Percent thigmotaxis during the acquisition phase for vehicle and CBD groups. **(d)** Percent time spent in the target quadrant and average of other three quadrants during the probe trial for vehicle and CBD groups. **(e)** Mean (mean ± SEM) number of annulus crossing during the probe trial for vehicle and CBD groups. **(f)** Mean (mean ± SEM) proximity during the probe trial for vehicle and CBD groups. *P < 0.05 and **P < 0.01, are considered statistically significant. a—as compared to day 1 in CBD group. b—as compared to day 1 in vehicle group. Vehicle group (*n* = 9; 7 male and 2 female), CBD group (*n* = 10; 8 male and 2 female).

The latency of aged mice to reach the hidden platform over eight training days is shown in Figure 4a. Both vehicle and CBD groups showed learning, which was confirmed by a repeated-measures ANOVA indicating a significant effect of days [*F*_(7,119)_ = 4.163, *P* < 0.01, $\eta_{\text{p}}^{2}$ = 0.197, observed power = 0.985]. No significant effects of group [*F*_(1,17)_ = 0.017, *P* = 0.899, $\eta_{\text{p}}^{2}$ = 0.001] and interaction [*F*_(7,119)_ = 1.360, *P* = 0.229, $\eta_{\text{p}}^{2}$ = 0.074] were observed. Although some learning occurred, the performance of both groups of aging subjects was impaired compared with younger mice [day *F*_(7,196)_ = 13.951, *P* < 0.001, $\eta_{\text{p}}^{2}$ = 0.333; group *F*_(2,28)_ = 9.618, *P* < 0.001, $\eta_{\text{p}}^{2}$ = 0.407; and their interaction *F*_(14,196)_ = 2.772, *P* < 0.001, $\eta_{\text{p}}^{2}$ = 0.165]. Young mice were not included in this study, but Supplementary Figure 1 shows the current MWT acquisition data compared with young mice that were run on the same task and training regimen in our laboratory. The swimming speed of mice over the eight training days is represented in Figure 4b, which shows that there is no difference in the swimming speed between both groups across training days. A repeated-measures ANOVA confirmed no significant effects of day [*F*_(7,119)_ = 1.204, *P* = 0.306, $\eta_{\text{p}}^{2}$ = 0.066], group [*F*_(1,17)_ = 0.042, *P* = 0.840, $\eta_{\text{p}}^{2}$ = 0.002], and their interaction [*F*_(7,119)_ = 0.410, *P* = 0.894, $\eta_{\text{p}}^{2}$ = 0.024]. The thigmotaxis behavior of mice during the eight training days is presented in Figure 4c. A repeated-measures ANOVA showed that both groups had significantly reduced thigmotaxis behavior as training progressed [*F*_(7,119)_ = 23.996, *P* < 0.001, $\eta_{\text{p}}^{2}$ = 0.585, observed power = 1.000]; however, no significant effects of group [*F*_(1,17)_ = 1.540, *P* = 0.232, $\eta_{\text{p}}^{2}$ = 0.083] and interaction [*F*_(7,119)_ = 0.625, *P* = 0.632, $\eta_{\text{p}}^{2}$ = 0.035] were observed.

The quadrant preference of both vehicle and CBD groups on the probe trial is represented in Figure 4d. A significant main effect of quadrant [*F*_(1,17)_ = 7.356, *P* = 0.015, $\eta_{\text{p}}^{2}$ = 0.302, observed power = 0.725] was observed, in which the CBD group spent significantly more time in the target quadrant rather than in the remaining quadrants compared with the vehicle group (*P* = 0.027). Annulus crossing on the probe day is shown in Figure 4e. Although the average annulus crossing on the probe trial was higher in the CBD group than in the vehicle group, there was no significant difference between them [t_(17)_ = −1.482, *P* = 0.157]. The average proximity of mice to the platform in the probe trial is represented in Figure 4f. No significant difference was observed in the proximity measure between vehicle and CBD groups [t_(17)_ = 1.260, *P* = 0.225].

### CBD had no effect on discriminative fear conditioning to context

**Figure 5:**
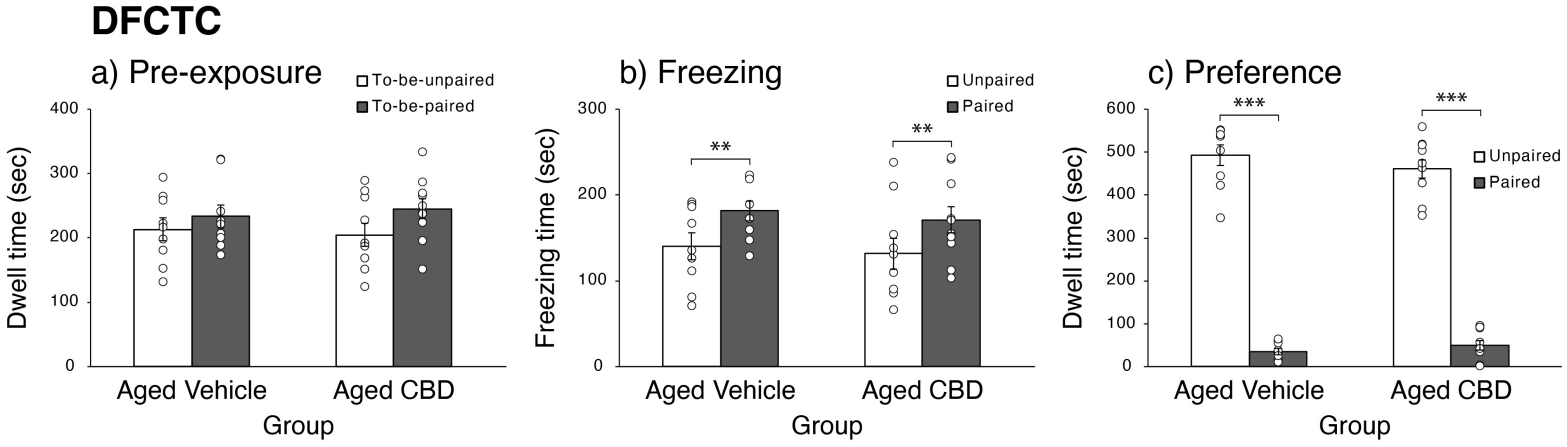
Effect of CBD on fear learning and memory function of 19-month-old C57BL/6 mice in discriminative fear conditioning to context task (DFCTC). **(a)** Mean dwell time (mean ± SEM) of mice during pre-exposure to contexts that were later assigned paired or unpaired in vehicle and CBD groups. **(b)** Mean freezing time (mean ± SEM) of mice in paired and unpaired contexts in vehicle and CBD groups. **(c)** Mean dwell time (mean ± SEM) of mice during the preference test in paired and unpaired contexts in vehicle and CBD groups. **P < 0.01 and ***P < 0.001 are considered statistically significant. Vehicle group (*n* = 9; 7 male and 2 female), CBD group (*n* = 10; 8 male and 2 female).

The results of the pre-exposure day are illustrated in Figure 5a, which shows that aged mice in vehicle and CBD groups spent approximately the same amount of time in each of the contexts. A repeated-measures ANOVA comparing time spent in the two contexts revealed no effects of context [*F*_(1,17)_ = 1.725, *P* = 0.207, $\eta_{\text{p}}^{2}$ = 0.092, observed power = 0.236], group [*F*_(1,17)_ = 0.041, *P* = 0.843, $\eta_{\text{p}}^{2}$ = 0.002, observed power = 0.054], and their interaction [*F*_(1,17)_ = 0.201, *P* = 0.660, $\eta_{\text{p}}^{2}$ = 0.012, observed power = 0.071]. During the freezing tests (Figure 5b), mice in the vehicle and CBD groups showed significantly more freezing behavior in the paired context than in the unpaired context, with no significant difference. This was confirmed by a repeated-measures ANOVA, in which there was a main effect of context [*F*_(1,17)_ = 11.860, *P* = 0.003, $\eta_{\text{p}}^{2}$ = 0.411, observed power = 0.900], but there was no effects of group [*F*_(1,17)_ = 0.275, *P* = 0.607, $\eta_{\text{p}}^{2}$ = 0.016] and their interaction [*F*_(1,17)_ = 0.006, *P* = 0.937, $\eta_{\text{p}}^{2}$ = 0.000]. As shown in Figure 5c, on the preference test day, mice in both groups preferred the unpaired context and spent the majority of the time there as compared to the paired context. This was confirmed by a repeated-measures ANOVA, in which there was a main effect of context [*F*_(1, 17)_ = 401.740, *P* < 0.001, $\eta_{\text{p}}^{2}$ = 0.959, observed power = 1.000], but there were no effects of group [*F*_(1,17)_ = 0.578, *P* = 0.458, $\eta_{\text{p}}^{2}$ = 0.033] and their interaction [*F*_(1,17)_ = 1.132, *P* = 0.302, $\eta_{\text{p}}^{2}$ = 0.062].

### No effect of CBD on HPC volume

**Figure 6:**
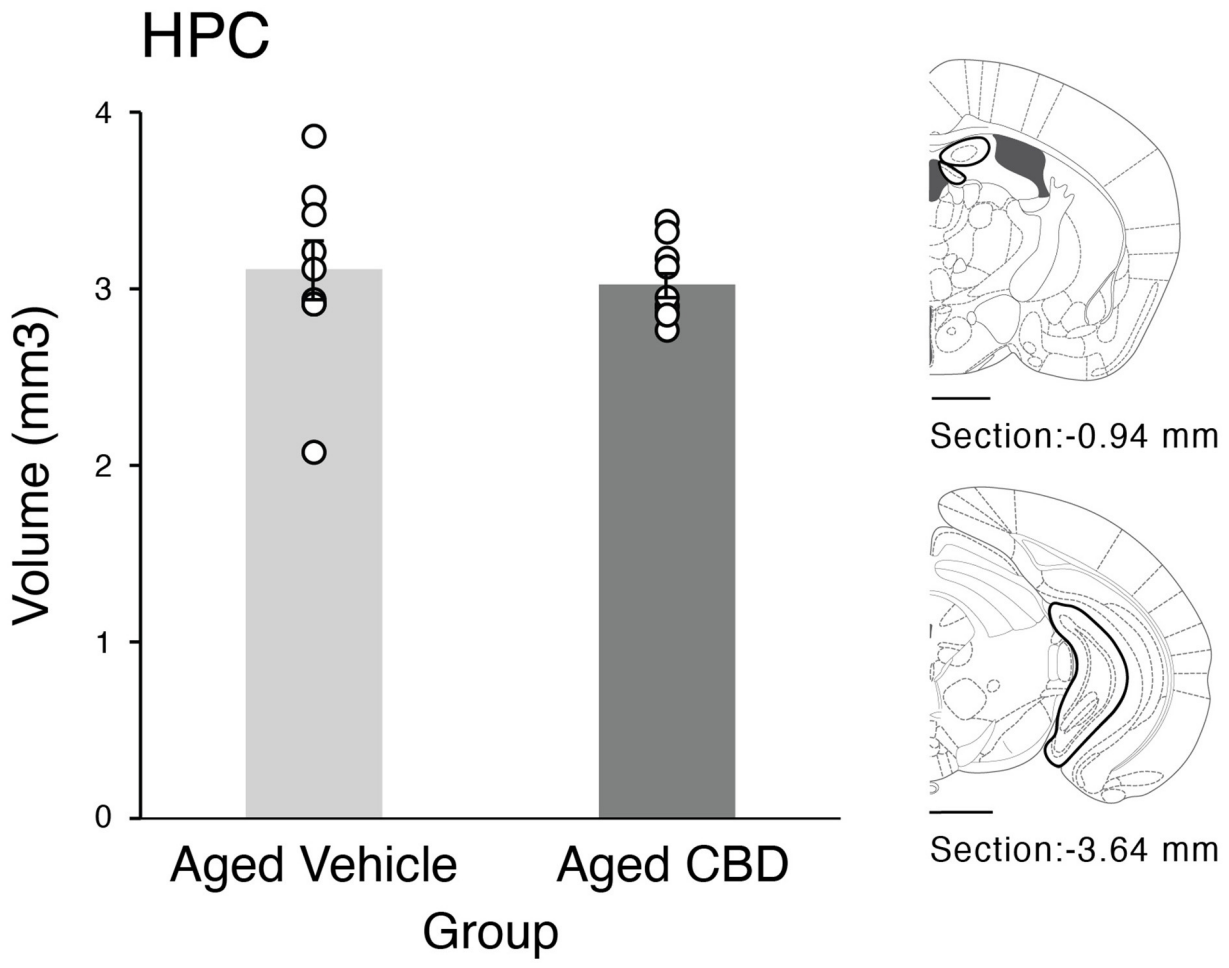
Effect of CBD on hippocampus volume in 21-month-old C57BL/6 mice. Hippocampal volume (mean ± SEM) in aged vehicle and CBD groups. Scale bars represent 1 mm. Vehicle group (*n* = 9; 7 male and 2 female), CBD group (*n* = 9; 7 male and 2 female).

The HPC volume in the vehicle and CBD groups is shown in Figure 6. There was no significant difference in the HPC volume of aged mice in vehicle and CBD groups, which was confirmed by an independent *t-*test [t_(16)_ = 0.394, *P* = 0.699, CI (−0.743, 1.109)].

### CBD decreased astrocytes in HPC

**Figure 7:**
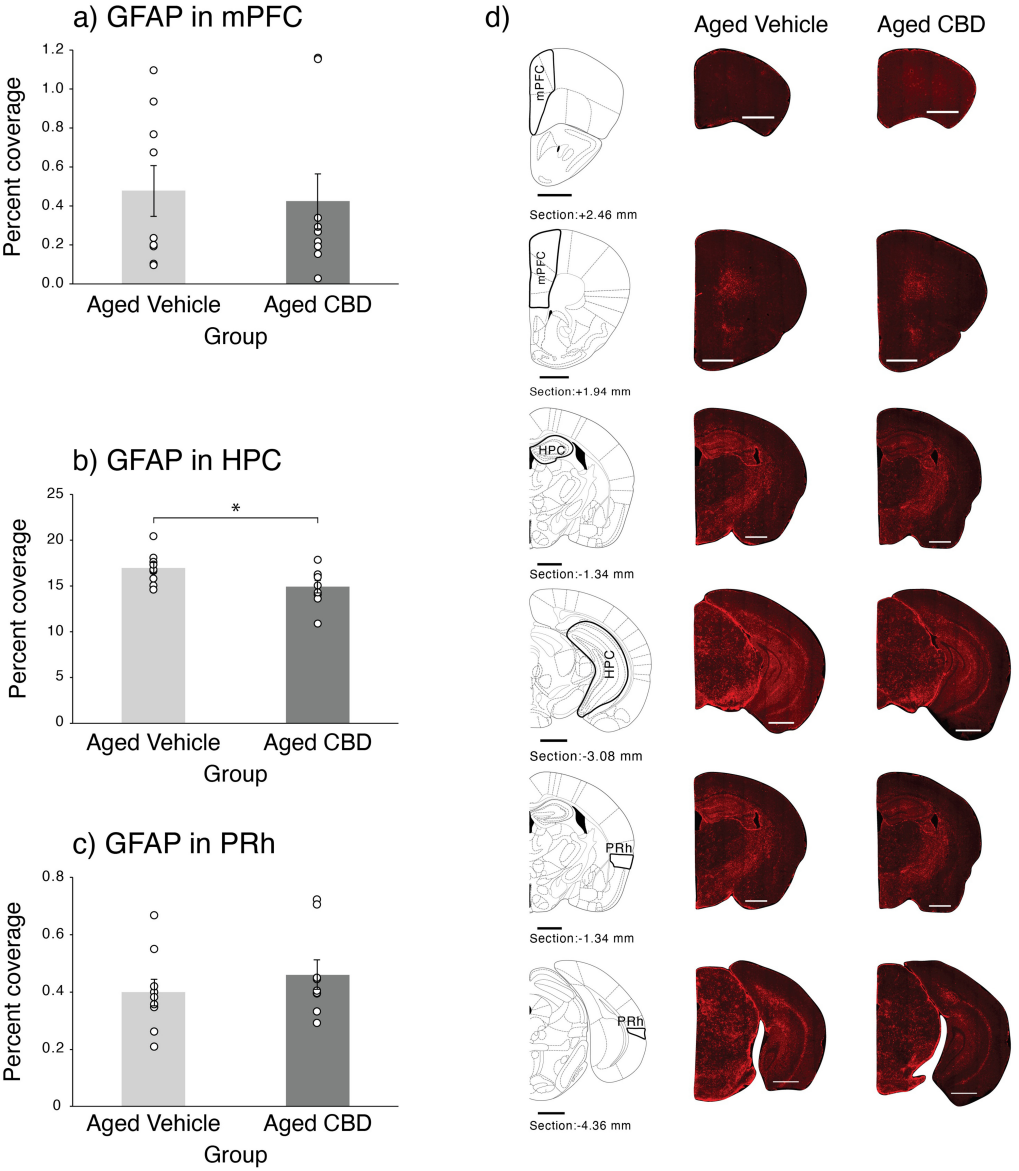
Effect of CBD on astrocytes in the mPFC, HPC, and PRh of 21-month-old C57BL/6 mice. **(a)** Percent coverage of GFAP in the mPFC (mean ± SEM) in vehicle and CBD groups. **(b)** Percent coverage of GFAP in the HPC (mean ± SEM) in vehicle and CBD groups. **(c)** Percent coverage of GFAP in the PRh (mean ± SEM) in vehicle and CBD groups. **(d)** Photomicrograph of GFAP in representative half slices for the mPFC, HPC, and PRh in aged vehicle and CBD-treated mice. Scale bars represent 1 mm. *P < 0.05 is considered statistically significant. Vehicle group (*n* = 9; 7 male and 2 female), CBD group (*n* = 9; 7 male and 2 female).

Astrocytes labeled by GFAP in the mPFC, HPC, and PRh in vehicle and CBD groups are shown in Figure 7. Aged mice that received CBD had decreased astrocytosis in their HPC, which was confirmed by an independent *t*-test [t_(16)_ = 2.303, *P* = 0.035, CI (0.075, 2.067)]. No significant difference was found in the mPFC and PRh.

### CBD decreased microglia in mPFC

**Figure 8:**
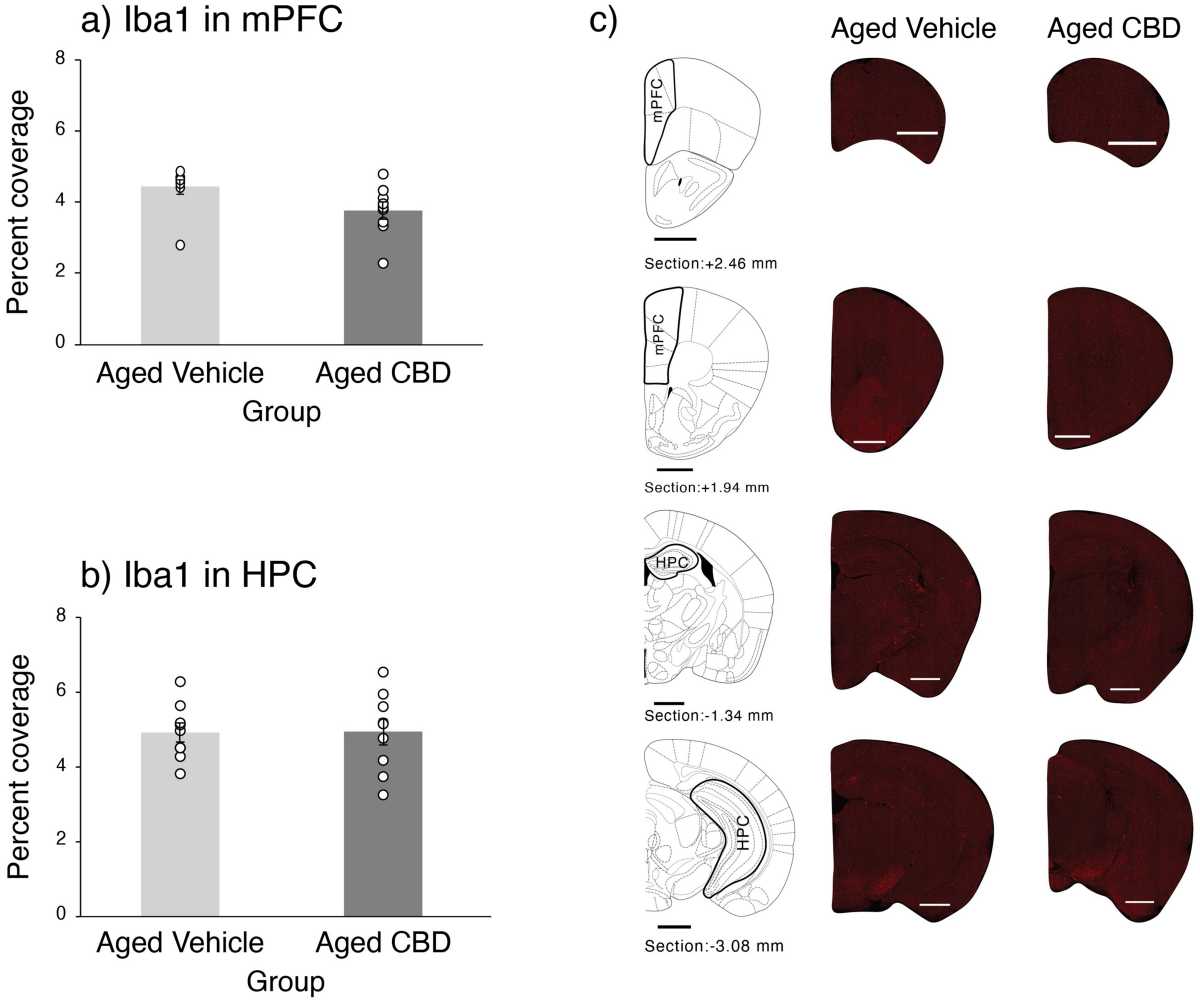
Effect of CBD on microglia in the mPFC and HPC of 21-month-old C57BL/6 mice. **(a)** Percent coverage of Iba1 in the mPFC (mean ± SEM) in vehicle and CBD groups. **(b)** Percent coverage of Iba1 in the HPC (mean ± SEM) in vehicle and CBD groups. **(c)** Photomicrograph of Iba1 in representative half slices for the mPFC and HPC in aged vehicle and CBD-treated mice. Scale bars represent 1 mm. Vehicle group (*n* = 9; 7 male and 2 female), CBD group (*n* = 9; 7 male and 2 female).

Microglia labeled by Iba1 in the mPFC and HPC in vehicle and CBD groups are shown in Figure 8. A marginally significant difference was found in the mPFC of aged mice as the mPFC of the CBD group had less percent coverage of microglia than that of the vehicle-treated mice [t_(16)_ = 2.098, *P* = 0.052, CI (−0.009, 1.960)]. No significant difference in microgliosis was found in the HPC between aged mice that received vehicle or CBD [t_(16)_ = −0.025, *P* = 0.981, CI (−0.935, 0.913)].

### CBD had no effect on ChAT

**Figure 9:**
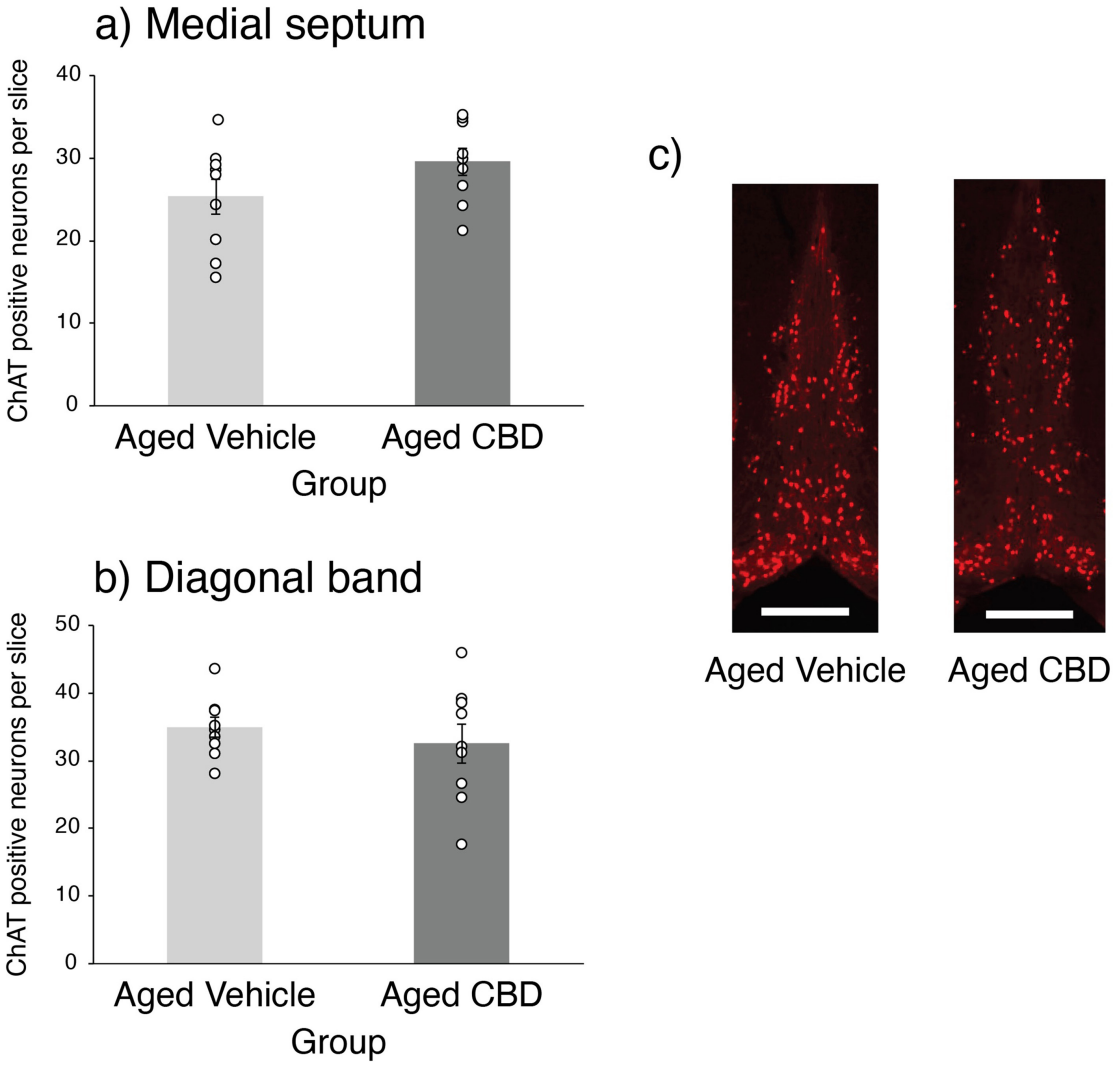
Effect of CBD on ChAT-positive neurons in the MS and DB basal forebrain of 21-month-old C57BL/6 mice. **(a)** Number of ChAT-positive neurons per slices in the MS (mean ± SEM) in vehicle and CBD groups. **(b)** Number of ChAT-positive neurons per slices in the DB (mean ± SEM) in vehicle and CBD groups. **(c)** Representative photomicrographs of ChAT-positive neurons in the MS and DB in aged vehicle and CBD-treated mice. Scale bar represents 500 μm. Vehicle group (*n* = 9; 7 male and 2 female), CBD group (*n* = 9; 7 male and 2 female).

The number of ChAT-positive neurons per slice labeled by ChAT IHC in the MS and DB in vehicle and CBD group is shown in Figure 9. No significant difference in the number of ChAT-positive neurons was observed in the MS and DB of aged mice in the vehicle and CBD groups, which was confirmed by an independent *t*-test [MS t _(16)_ = −1.584, *P* = 0.133, CI (−1.695, 0.223); DB t _(16)_ = 0.725, *P* = 0.479, CI (−0.595, 1.268)].

## Discussion

This study assessed the effect of a cannabinoid treatment on various brain regions associated with learning and memory and the related brain pathology in aged C57 mice. Fourteen-month-old mice were administered vehicle or CBD orally daily for 7 months. At 19 months of age, behavioral testing was started using NOR, BB, MWT, and DFCTC. After behavioral testing was completed, mice were perfused at 21 months of age for histological assessment. Nissl staining was used to measure HPC cell volume, and IHC analysis was carried out using GFAP, Iba1, and ChAT. The results indicated that aged mice treated with CBD showed improved performance on the NOR and MWT, suggesting that CBD treatment has a positive impact on object memory processes mediated by the PRh and spatial memory functions centered on the HPC. Brain pathology results showed that the aged mice treated with CBD showed reduced inflammation in the HPC and mPFC, but not in the PRh. There was no indication that CBD differentially affected the pathology of the mice depending on sex; however, the number of female subjects in this study was limited. Furthermore, long-term administration of CBD did not negatively affect learning and memory functions, or the brain of the aged subjects, based on our suite of measures. Taken together, these results suggest that oral CBD treatments can improve memory processes that are impaired due to aging. Brain regions and associated functions are mediated by structures in the medial temporal lobe that are typically compromised in age-related cognitive decline, and inflammatory responses in some of these brain regions were also reduced. Importantly, evidence was also observed suggesting that long-term CBD treatments do not have negative impacts on these same functions and brain regions, nor do they cause negative side effects in aged mice subjects.

### Perirhinal cortex and object memory processes

One of the key studies conducted on rats and monkeys has suggested that the PRh is important for recognition memory (Kealy and Commins, 2011). Anatomical and electrophysiological evidence is consistent with this finding as the PRh receives and processes extensive visual and olfactory input from the neocortex (Burwell, 2000) and seems crucial for visual and olfactory recognition memory functions (Brown et al., 2010; Buckley et al., 2001; Gaffan, 1994; Hannesson et al., 2005; Kaut and Bunsey, 2001; Kaut et al., 2003; Kealy and Commins, 2011; Mumby et al., 2002; Mumby and Pinel, 1994; Mumby et al., 2007; Suzuki, 1996). Another study has suggested that the specific role of the PRh lies in one aspect of recognition memory: familiarity detection, not recollection (Brown and Aggleton, 2001). In aged rats, deficits in PRh-dependent behavioral tasks are evident, with a marked reduction in the firing rate activity of different cell types within this region while rats learn a novel object task. This reduction may lead to a reduced focus on details and an overemphasis on processing general or gist-like information (Maurer et al., 2017). This impairment is also observed in aged primates (Burke et al., 2011) and humans (Ryan et al., 2012).

### Hippocampus and spatial memory processes

Half a century of evidence converging from different fields is consistent with the notion that the HPC is a central structure of a spatial/relational learning and memory network (Eichenbaum et al., 1988; Hirsh, 1974; O'Keefe and Nadel, 1978). The HPC seems essential when an organism must learn about relationships between cues to solve a particular task. For example, rats with HPC damage have been reported to be impaired at the acquisition and retention of various spatial tasks such as the MWT (Morris et al., 1982; Sutherland et al., 1982), a finding that is reliable and valid to date (McDonald and Hong, 2000; McDonald et al., 2004).

In the present study, mice in the CBD and vehicle groups did not show normal learning curves or low asymptotic levels of performance like young adult mice. To further explain this finding, we included a young adult mouse group from another study to show the normal pattern of learning on this task (Supplementary Figure 1). Clearly, the aged subjects in both groups were impaired in the acquisition of the task although they did show some limited improvements. However, during the probe trial, only the CBD-treated mice spent more time swimming in the target quadrant. These results suggest that the CBD treatment has some impact on the ability of the aged mice to remember the spatial location 24 h later.

The fact that neither the acquisition of the task nor measures of spatial specificity (annulus crossings or proximity to escape location) were affected suggests that some general spatial navigation process was improved in the CBD-treated mice. One possibility is that the treatment targeted the ventral HPC. This sub-region of the HPC is presumed to be involved in getting the subject to the general goal area, and these experiences with the escape platform would result in the formation of a precise spatial representation of the exact goal location in the dorsal HPC, leading to spatial specificity (Gruber and McDonald, 2012). Consistent with this idea, both dorsal and ventral HPC contribute to the acquisition of spatial information though the former plays a more prominent role in spatial learning and navigation (Ferbinteanu et al., 2003) and has been shown to be required for spatial specificity (McDonald et al., 2018; Ruediger et al., 2012). These analyses suggest that the CBD treatment acts on the ventral HPC specifically. However, further work is necessary to confirm this observation.

Finally, it is interesting to note that the aged model used in present experiments did not show depletions of cholinergic neurons in the MS/DB; projections from these neurons provide the HPC with extensive acetylcholine tone. These depletions are observed in human AD patients and other preclinical rodent models of AD (Craig et al., 2011; Mehla et al., 2023, 2022) and can be attributable to certain treatment interventions. This pattern suggests a relationship between protein malformations associated with AD and cholinergic depletions independent of aging.

### Modes of action of cannabinoids on the brain

Cannabinoids exert their effects on the brain primarily by interacting with the endocannabinoid system, a complex cell-signaling network that regulates various physiological processes. This system includes two primary receptors, CB1 and CB2, though cannabinoids can also influence other receptors and pathways indirectly. THC, the main psychoactive component of cannabis, directly activates both CB1 and CB2 receptors in the brain, while CBD, which is non-psychotomimetic, acts on these receptors indirectly. CB1 receptor activation primarily modulates neuronal function, whereas CB2 activation influences immune cells such as microglia (Benito et al., 2008; Burstein, 2015; Nagarkatti et al., 2009; Pertwee, 2008).

In addition to cannabinoids, terpenoids play a key role in the therapeutic effects of cannabis and contribute to its unique aroma and the associated entourage effect. The entourage effect indicates that cannabinoids and terpenes act synergistically to produce effects that are more potent or distinct than when the compounds are used in isolation (Ben-Shabat et al., 1998; Ferber et al., 2020). Research has shown that cannabis extracts that contain a mixture of cannabinoids and terpenes are more effective at reducing inflammation than CBD or THC alone (Russo, 2011). Therefore, we hypothesize that an extract containing CBD, THC, and terpenoids would produce more effective therapeutic effects. Future research needs to examine the effects of such extracts on the brain.

### Cannabinoid treatment efficacy might depend on the severity of neurodegenerative disease

The efficacy of cannabinoid treatment remains a topic of ongoing research, largely due to the complex interactions between cannabinoids and the endocannabinoid system. *In vitro* studies have suggested that repeated activation of CB1 and CB2 receptors can reduce brain pathology and cognitive deficits associated with AD by decreasing Aβ levels, inflammation, oxidative stress, excitotoxicity, and ischemia (Benito et al., 2008; Paloczi et al., 2018; Ramírez et al., 2005). Similarly, *in vivo* studies in the APP × PS1 mouse model of AD have shown improvements in social and object recognition, but no effect on Aβ levels (Cheng et al., 2014). However, several studies conducted in our laboratory have found little to no impact on AD progression. Specifically, investigations using different dosages of THC and CBD in the APP-NL-G-F mouse model of AD have revealed no significant effect on memory-related behavioral tasks or inflammation (Nixon et al., 2022), and a combination of cannabis extract used to improve pathology or memory deficits in the same mouse model has also shown no effect (Robinson et al., 2023). Based on the results of the present experiments, it seems that the severity and aggressiveness of the type of age-related cognitive decline determine whether cannabinoids, or any treatment, might be effective or not.

### Inflammation and age-related cognitive decline

There is a well-established association between inflammation and age-related dementia. As people grow older, the immune system undergoes changes that lead to a state of chronic inflammation, often referred to as “inflammaging” (Franceschi and Campisi, 2014). This state is characterized by increased levels of pro-inflammatory markers in the bloodstream and tissues. Neuroinflammation is a hallmark of many forms of dementia and involves the activation of microglia and astrocytes, which can become overactive in response to injury or disease (Franceschi and Campisi, 2014). The findings of the present study suggest that chronic CBD treatment in aging mitigates astrocyte-mediated inflammation, probably by modulating astrocytic signaling pathways. One proposed mechanism is that CBD inhibits equilibrative nucleoside transporter 1 (ENT1), which is responsible for the uptake of adenosine from the extracellular space into cells (Carrier et al., 2006; Ibeas Bih et al., 2015). By inhibiting ENT1, CBD increases the extracellular concentration of adenosine, which results in enhanced interaction with adenosine receptors on astrocytes. This interaction in turn activates adenylate cyclase and subsequently protein kinase A. These signaling events result in the downregulation of pro-inflammatory cytokines and other inflammatory mediators, thereby reducing inflammation. However, further studies are required to comprehensively understand molecular signaling pathways and to identify potential common or distinct mechanisms by which CBD regulates inflammation and synaptic functions.

### Other potential targets of CBD

Emerging evidence from animal studies suggests that CBD promotes adult neurogenesis, particularly in the HPC, by enhancing the survival of newborn neurons in the dentate gyrus (Wolf et al., 2010) and by interacting with the nuclear transcription factor PPARγ, which increases neurogenic activity (Esposito et al., 2011). Moreover, CBD has been shown to modulate neurotransmitter systems such as upregulating norepinephrine (Abame et al., 2021). These effects highlight the potential of CBD as a promising therapeutic target for future research, particularly in addressing age-related dementia.

### Caveats

One caveat associated with our study is that it primarily used male mice, with only a small number of female subjects, which limits our conclusions on potential sex-dependent effects of CBD. This disparity in the number of female and male subjects occurred because we used mice that were available at the time of the study; due to unpredictable litter size and sex distribution, the inclusion of a small number of female mice was unavoidable. To address concerns regarding potential sex-dependent effects, we carefully investigated the raw data from female subjects, and their values were well within the range of the male subjects. Thus, the inclusion of only two female mice per group did not affect our primary conclusions and allowed us to modestly increase our overall sample size.

Another caveat is the mode of administration of CBD. It has been reported that oral administration may not be the most effective method for optimal bioavailability and brain penetration. Although oral CBD delivery may have lower bioavailability than other routes such as intravenous administration, it remains a practical and widely used method, particularly in translational research. Marusich and Wiley (2023) found that the THC administration route has minimal impact on potency and duration of effect in C57 mice (except for aerosolized delivery) although THC and CBD have distinct pharmacokinetic properties. In addition, daily intraperitoneal or subcutaneous injections for 7 months would have been impractical due to stress-related effects on the animals and the increased risk of injection-related variability. Thus, oral administration was chosen for its feasibility, translational relevance, ability to minimize procedural stress, and consistency in treatment administration across experimenters.

Finally, the length of the treatment used in our study might be considered unrealistic. The daily CBD treatment lasted 7 months, which is equivalent to 10–20 years of human life. Our aim was to evaluate the long-term effects of CBD supplementation in an attempt to understand the preventative potential of CBD over a considerable part of the animal's lifespan rather than a short-term therapeutic intervention. Given that preventative strategies such as dietary and vitamin supplements in humans are frequently taken for many years or even lifelong, we believe our approach realistically reflects how CBD might be employed in a human preventative context.

## Conclusion

The findings of this study show that CBD targets inflammatory responses in the brain and can improve cognitive decline associated with aging. It is possible that the effects of CBD treatment can be enhanced if an extract with THC and terpenoids is used.

## Data Availability

The original contributions presented in the study are included in the article/Supplementary material, further inquiries can be directed to the corresponding authors.

## Supplementary material

Supplementary Figure 1 Effect of CBD on spatial learning and memory function of 19-month-old C57BL/6 mice in MWT task compared with 3-months old C57BL/6 mice from another study conducted in our lab. ^**^*P* < 0.01 is considered statistically significant. a—aged CBD group Day 8 compared to Day 8 of 3-months old mice. b—aged Vehicle group Day 8 compared to Day 8 of 3-months old mice.
