# Supplementary figures and images for: Effects of cannabidiol (CBD) treatment on age-related cognitive decline in C57 mice
Source: Front Aging Neurosci. 2025 May 9;17:1567650. doi: 10.3389/fnagi.2025.1567650 (PMC12098523; doi:10.3389/fnagi.2025.1567650)

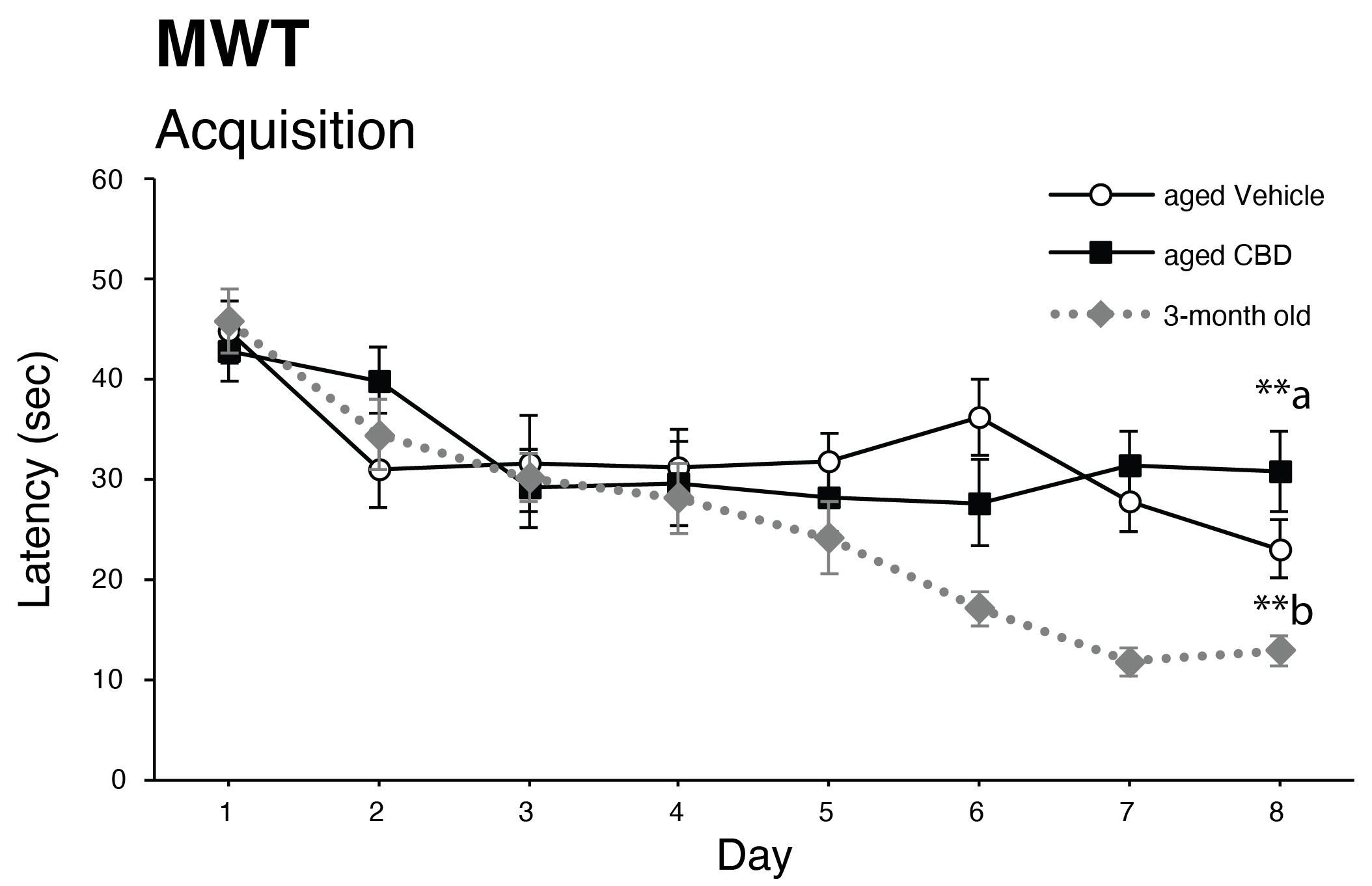

Supplement: Supplementary Figure 1 — Effect of CBD on spatial learning and memory function of 19-month-old C57BL/6 mice in MWT task compared with 3-months old C57BL/6 mice from another study conducted in our lab. **P < 0.01 is considered statistically significant. a—aged CBD group Day 8 compared to Day 8 of 3-months old mice. b—aged Vehicle group Day 8 compared to Day 8 of 3-months old mice. [file Image_1.jpg]
